# Evidence for population variation in *TSC1 *and *TSC2 *gene expression

**DOI:** 10.1186/1471-2350-12-29

**Published:** 2011-02-23

**Authors:** Garilyn M Jentarra, Stephen G Rice, Shannon Olfers, David Saffen, Vinodh Narayanan

**Affiliations:** 1 Neurology Research Department, Barrow Neurological Institute, 350 W. Thomas Rd., NRC 438, Phoenix, AZ, 85013, USA; 2 Institutes of Brain Science, Fudan University, 1015 Mingdao Hall, 138 Yixueyuan Rd, Shanghai 200032, PR China; 3 School of Life Sciences, Arizona State University, University Drive and Mill Ave., Tempe, AZ, 85287, USA

## Abstract

**Background:**

Tuberous sclerosis complex (TSC) is an autosomal dominant neurogenetic disorder caused by mutations in one of two genes, *TSC1 *or *TSC2*, which encode the proteins hamartin and tuberin, respectively [1-3]. Common features of TSC include intractable epilepsy, mental retardation, and autistic features. TSC is associated with specific brain lesions, including cortical tubers, subependymal nodules and subependymal giant cell astrocytomas. In addition, this disease frequently produces characteristic tumors, termed hamartomas, in the kidneys, heart, skin, retina, and lungs. Disease severity in TSC can be quite variable and is not determined by the primary mutation alone. In fact, there is often considerable variability in phenotype within single families, where all affected individuals carry the same mutation. Factors suspected to influence phenotype in TSC include the specific primary mutation, random occurrence of second-hit somatic mutations, mosaicism, "modifying genes", and environmental factors. In addition to these factors, we hypothesize that differences in mRNA expression from the non-mutated TSC allele, or possibly from the mutated allele, play a part in modifying disease severity. Common genetic variants that regulate mRNA expression have previously been shown to play important roles in human phenotypic variability, including disease susceptibility. A prediction based on this idea is that common regulatory variants that influence disease severity in TSC should be detectable in non-affected individuals.

**Methods:**

A PCR/primer extension assay was used to measure allele specific expression of *TSC1 *and *TSC2 *mRNAs in leukocytes isolated from normal volunteers. This assay can be used to measure "allelic expression imbalance" (AEI) in individuals by making use of heterozygous "marker" single nucleotide polymorphisms (SNPs) located within their mRNA.

**Results:**

In this study we show for the first time that *TSC1 *and *TSC2 *genes exhibit allele-specific differences in mRNA expression in blood leukocytes isolated from normal individuals.

**Conclusions:**

These results support the possibility that allele-specific variation in *TSC *mRNA expression contributes to the variable severity of symptoms in TSC patients.

## Background

Tuberous sclerosis complex (TSC) is an autosomal dominant neurogenetic disease caused by a mutation in either the *TSC1 *or *TSC2 *gene [1-3]. Roughly two-thirds of TSC cases reported in mutational and epidemiological studies are sporadic (simplex), while the remaining cases are familial [4-9]. Neurological symptoms include seizures, cognitive delay, impulsivity, attention deficit, and learning disabilities. TSC patients often present with characteristic brain lesions, including cortical tubers, subependymal nodules (SENs), and subependymal giant cell astrocytomas (SEGAs). The severity of neurological symptoms is variable, although mental retardation and intractable epilepsy are fairly common and are frequently the most debilitating symptoms [2,10,11].

Lesions outside of the nervous system, including renal angiomyolipomas (AMLs), renal cysts, cardiac rhabdomyomas, facial angiofibromas, periungual fibromas, retinal hamartomas, and pulmonary lymphangioleiomyomas (LAM), are also characteristic of TSC [2,11]. Some of these lesions may result in life threatening events, such as hemorrhage into a large AML [12,13] or spontaneous pneumothorax or chylothorax from a ruptured LAM [14].

Many of the hamartomatous growths associated with TSC are likely to be caused by loss of heterozygosity (LOH) due to a "second-hit" mutation that compromises the remaining normal TSC allele. This has been demonstrated in renal AMLs, cardiac rhabdomyomas, SEGAs and SENs [13,15-18]. By contrast, LOH has only rarely been demonstrated in cortical tubers [19,20]. While the lesions of TSC are generally associated with LOH, cognitive symptoms, including mental retardation, hyperactivity, impulsivity and attention deficit, may occur by a different mechanism, likely involving haploinsufficiency of TSC proteins in brain cells. In fact, the pathway in which hamartin and tuberin function has been shown to influence both neuronal structure and function [21]. It is therefore plausible that dysregulation of this pathway (a quantitative effect) produces cognitive deficits.

Studies of coding and splice region mutations of the TSC1 and TSC2 genes have not yet produced a clear understanding of the relationship between genotype and phenotype, as people with the same primary mutation often have very different phenotypic outcomes [22,23]. It is generally accepted, however, that mutations in the TSC1 gene produce milder symptoms compared to mutations in the TSC2 gene [4,5,9,24]. Although most studies have failed to consistently link specific mutations to distinct phenotypes, there are exceptions such as the TSC2 R905Q mutation, which produces a mild form of the disease, and the TSC2 R905W and R905G mutations, which are associated with more severe forms of TSC [25].

Our research is aimed at understanding why individuals carrying identical TSC gene mutations often have widely varying clinical outcomes. It has been repeatedly noted in the literature that phenotypic variation of TSC disease is very common within families [2,26-31]. The reason for this intra-familial variability in phenotype is currently unknown, although potential explanations include the modifying effects of unlinked genes, epigenetic factors [32,33], or mosaicism [34,35].

In many simple genetic disorders, pathogenic mutations inactivate the encoded protein or reduce its quantity or stability, thereby leading to an inadequate level of functional protein in the cell. TSC is an autosomal dominant genetic disease and, consequently, affected individuals are heterozygous for mutations in TSC1 or TSC2, i.e., one mutant and one normal allele is present in each cell [1-3]. We hypothesize that the differential expression of normal and mutant alleles may account for some proportion of the observed phenotypic variation. For example, it is possible that at the cellular level, relatively high levels of expression of the normal allele may compensate for the abnormal protein produced by the mutant allele. Conversely, high expression of a "gain of function" mutant protein, such as a mutant with dominant-negative properties, may be particularly deleterious. Based upon these considerations, it is plausible that allele-specific *cis*-acting elements that regulate mRNA expression [36-39] contribute to differences in disease severity in TSC.

If common regulatory elements within the TSC loci play a role in modulating disease phenotype in individuals carrying a mutation at one of the TSC genes, we would expect to be able to detect the same regulatory elements in subjects selected from the normal population. To test this hypothesis we used a PCR/primer extension-based assay to measure allele-specific differences in expression of *TSC1 *and *TSC2 *mRNAs in leukocytes isolated from normal volunteers. The use of this assay allows highly accurate measurements of "allelic expression imbalance" (AEI) for individuals who are heterozygous for a "marker" single nucleotide polymorphism (SNP) located within the mRNA. Based on these measurements, we estimate that about 19% of the population (our sample group was of mixed races with a predominance of Caucasian individuals) is heterozygous for high- and low-expression alleles at the *TSC1 *locus and 10% of the population is heterozygous for high- and low-expression alleles at the *TSC2 *locus.

## Methods

### IRB Approval

This research was approved by the St. Joseph's Hospital and Medical Center Institutional Review Board (IRB) for Human Research. Informed consent was obtained from all study participants. Participants were healthy volunteers who denied any personal or familial history of Tuberous Sclerosis Complex.

### Isolation of DNA and RNA from blood samples

DNA was extracted from blood leukocytes using Gentra Puregene Blood Kits (Qiagen, Valencia, California) and stored at 4°C. RNA was extracted from blood leukocytes using PAXgene Blood RNA Kits (Qiagen, Valencia, California) and stored at -80°C.

### cDNA Synthesis from RNA samples

*TSC1 *(NM_000368) and *TSC2 *(NM_000548) mRNAs were reverse-transcribed to cDNA using gene-specific primers and the SuperScript III First-Strand synthesis system for RT-PCR, according to the manufacturer's protocol (Invitrogen, Carlsbad, California). The cDNA synthesis primer sequence for *TSC1 *mRNA was 5'-GGGCCTGTGCTGACTCTGGTTAGTG-3'. The sequence of the cDNA synthesis primer for *TSC2 *mRNA was 5'-TTTCACTGACAGGCAATACC-3'. cDNAs were stored at -20°C.

### Selection of Coding Region SNPs in TSC1 and TSC2

To distinguish TSC gene alleles we chose marker SNPs with relatively high rates of heterozygosity, as indicated in the NCBI Human Genome Resource SNP database http://www.ncbi.nlm.nih.gov, the SNPper resource (CHIP Bioinformatics resource - http://snpper.chip.org) and by our own genotyping data. Due to the need to distinguish the alleles, only samples heterozygous at marker SNPs were analyzed. Two SNPs were chosen as markers for *TSC1 *alleles: rs739442 (C/T) and rs2809243 (C/T), both located in the 3'-untranslated region (UTR) of *TSC1 *mRNA. The DNA samples were genotyped at several exonic SNPs in the TSC2 gene. One synonymous SNP located within exon 40, rs1748 (C/T), proved to have the highest rate of heterozygosity among the tested SNPs (~24%) and was therefore used for AEI analysis. Together, these three marker SNPs tag all known *TSC1 *and *TSC2 *mRNA splice variants.

### Genotyping using the SNaPshot assay

All samples were genotyped using the SNaPshot assay. This method of genotyping relies on the presence of heterozygous marker SNPs to distinguish between two alleles of a gene. In homozygous samples, where the gene alleles have the same nucleotide at the SNP locus, electropherograms will show a single peak using the forward primer and a single peak using the reverse primer. In heterozygous samples, the presence of different nucleotides at the SNP locus on each allele will result in the production of two peaks in both forward and reverse reactions.

PCR primer pairs were designed for amplifying genomic DNA segments that included each SNP of interest. The amplimer segments were used in a SNaPshot assay (ABI Prism SNaPshot Multiplex Kit) to establish the genotype (homozygous versus heterozygous) of individuals at each of the marker SNPs. The primers for amplifying the 3'UTR genomic DNA segment containing the *TSC1 *SNPs rs2809243 and rs739442 (amplimer size = 587 bp) were: 5'-TAGTAATGGCAGAGCAGTCTAAACA-3' (forward) and 5'-TCCAGGTCTCATTCTCCCAACCGTA-3' (reverse). The primers for amplifying a genomic DNA segment surrounding TSC2 exon 40 were: 5'-CTGGGCAACGACTTTGTGTCCATTGTCTAC-3' (forward) and 5'-CTGACAGGCAATACCGTCCAA-3' (reverse). This primer pair produces an 1857 bp amplimer when used with genomic DNA. The PCR program consisted of an initial denaturation at 94°C for 40 seconds. This was followed by 40 cycles of 94°C for 20 seconds, 55°C for 1 minute, 72°C for 1 1/2 minutes, and a final extension step at 72°C for 5 minutes. PCR products were gel purified from 1-1.5% low melt agarose gels (IBI Scientific, Peosta, Iowa) using the Wizard PCR Preps DNA Purification System (Promega, Madison, Wisconsin).

Genotyping was done with primers designed for SNaPshot analysis. The SNaPshot assay was performed according to the manufacturer's protocol (Applied Biosciences, Inc.). Briefly, a PCR reaction was run in which a single fluorescently-tagged dideoxynucleotide was added at the 3'-end of an annealed primer that was designed to terminate exactly one nucleotide before the SNP of interest (different fluorophores for ddA, ddG, ddC, and ddT). This allowed the identity of the SNP nucleotide to be determined using a capillary sequencer (Applied Biosystems Inc. Prism 310 Genetic Analyzer). This assay was used both for genotyping individuals at various SNPs and for AEI determination (as described in the following section). While both forward and reverse primers can be used for this analysis, the forward primers for each of the SNPs analyzed were found to give cleaner, more reliable results and were thus used in this assay.

The primers for SNaPshot analysis of the TSC1 gene alleles were: rs2809243 5'-AAACTCAACAAGTGCTCCTGAAAGAAA-3' (forward) and rs739442 5'-TACGAAATCTTAGTGCC-3' (forward). The primer for SNaPshot analysis of the TSC2 gene allele was rs1748 (forward): 5'-GCATCATAGCCGCTCCAACCCCACCGA-3'. The PCR program consisted of 25 cycles of a 96°C denaturation step for 10 seconds, 50°C for 5 seconds and 60°C for 30 seconds. Subsequently, samples were treated for 45 minutes at 37°C with 5 units of antarctic phosphatase (New England Biolabs, Ipswich, Massachusetts). The phosphatase was then inactivated by incubating at 65°C for 10 minutes. Samples were run on the capillary sequencer and the genotype determined from the electropherogram generated during the run.

### Allelic expression imbalance assay

Samples heterozygous at marker SNPs were tested for AEI using the SNaPshot assay. Genomic and cDNA fragments flanking each SNP (as described above) were amplified in triplicate and gel purified. The same primers previously described for use in the amplification of genomic DNA segments were used in this assay to amplify TSC1 and TSC2 cDNA gene segments. The primer pair amplifying the TSC2 cDNA segment produces a 553 bp fragment when used with cDNA rather than the 1857 bp fragment produced with genomic DNA as the template. This is due to inclusion of intronic sequence in the PCR product from genomic DNA. The PCR reactions amplify both alleles, preserving the existing allele ratios in genomic DNA and in cDNA. The concentrations of gel purified samples (purification performed as indicated above) were measured using a Nanodrop 2000c (Thermo Scientific, Waltham, Massachusetts). Equal concentrations of the amplified fragments were then used in SNaPshot assays. All genomic and cDNA samples were analyzed in triplicate using the ABI capillary sequencer.

**Figure 1:**
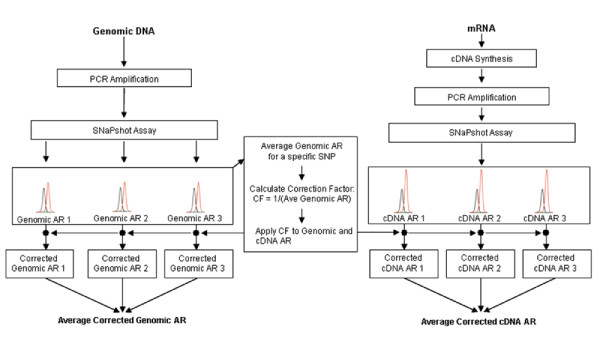
**Method for correcting genomic and cDNA allele ratios (AR)**. Genomic DNA segments containing marker SNPs are amplified by PCR and used as templates in SNaPshot primer-extension assays. Extended primers containing one of two different fluorescently labeled nucleotides at their 3'-ends are resolved by capillary electrophoresis and the ratio of peak heights calculated. An average genomic AR for a specific SNP is determined from all the genomic samples (each analyzed in triplicate). A correction factor (CF) is then calculated as the inverse of the average genomic AR. The genomic samples analyzed in triplicate are then individually multiplied by the CF to normalize the data to approximately 1.0, which is the theoretical ratio of two gene alleles in genomic DNA. The corrected average genomic AR is then determined. For each RNA sample, cDNA is synthesized and heterozygous SNP containing segments are amplified by PCR in triplicate and subjected to a SNaPshot PCR reaction. Samples are run on a capillary sequencer and the ratio of heterozygous peak heights is determined. Individual cDNA ARs are calculated and corrected by multiplying by the CF. The average corrected cDNA AR for each sample is then calculated. A sample is designated as showing AEI if the average corrected genomic AR and average corrected cDNA AR differ by greater than 2X the standard error of the mean and by at least 10%.

Genomic DNA has a theoretical allele ratio of 1.0, but due to differences in the detection efficiency of various fluorophores, this ratio often deviates from 1.0. Therefore, genomic DNA was used as an internal control to correct for the differences in detection. In order to calculate the necessary correction factor, the genomic DNA allelic ratios for a specific SNP from each capillary sequencer run were averaged and the correction factor was calculated as the inverse of this average genomic allelic ratio. A diagram of the method used for calculating and applying the correction factor is shown in Figure 1. The allele ratios for genomic and cDNA samples were calculated as the ratio of heterozygous peak heights (analysis done using Gene Mapper 3.0 software from Applied Biosystems, Inc.). The experimental values for both the genomic DNA and the cDNA were then multiplied by the correction factor and average values were calculated for each sample analyzed in triplicate (see Results section for additional details). Standard error of the mean (SEM) was calculated for each sample using Excel software (Microsoft, Inc.) and error bars indicating 2X SEM were used in graphing the results.

## Results

**Figure 2:**
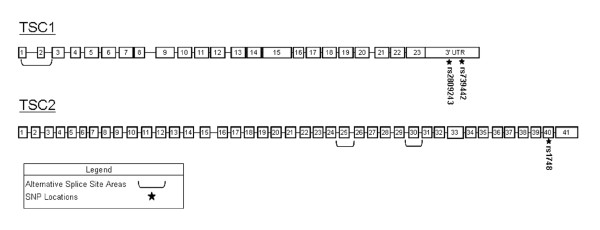
**This diagram shows the exon/intron structure of the *TSC1 *and *TSC2 *genes**. Exons are represented by numbered boxes. Exons subject to alternative splicing are indicated by brackets. The locations of the SNPs used for analysis of AEI are indicated by stars.

AEI was examined in the *TSC1 *and *TSC2 *genes by quantifying the relative amounts of mRNA derived from each of the two alleles of each gene in leukocyte RNA samples isolated from normal individuals heterozygous for mRNA marker SNPs. To avoid the confounding effects of alternative splicing, SNPs located within the 3'-UTR of the *TSC1 *gene and in exon 40 of the *TSC2 *gene were selected as markers. These regions are included in all known mRNA forms generated from the *TSC1 *and *TSC2 *genes. The rs numbers (NCBI SNP data base, http://www.ncbi.nlm.nih.gov/snp) and locations of the three *TSC1 *and *TSC2 *marker SNPs used in this study are shown in Figure 2.

As described in detail in Methods, our AEI assays involve PCR amplification of short segments of *TSC1 *or *TSC2 *cDNA containing a marker SNP, followed by annealing of a synthetic oligonucleotide primer to a site immediately upstream from the SNP and primer extension in the presence of fluorescently tagged dideoxynucleotide triphosphates (ddNTPs). Because each ddNTP is tagged with a different fluorophore, the identity of the added base can be determined by resolving the fluorescently labeled primers by capillary electrophoresis and identifying the "color" of each extended primer [40].

Differences in expression between two alleles can be quantified by calculating the ratio of the peak heights of the traces corresponding to each fluorescently labeled primer. To correct for artifactual imbalances related to technical aspects of the assay, AEI assays were also carried out using genomic DNA, which in the absence of local chromosome deletions or duplications, would be expected to contain equal numbers of each allele. A correction factor derived from these measurements was used to correct AEI measurements obtained using cDNA templates.

### SNP frequencies in the sample population

Our assay uses SNPs located within protein coding exons or the 3'-UTR to distinguish between the mRNA species that are transcribed from the two alleles of a gene in each individual. Because only subjects who are heterozygous at marker SNPs are informative in our assays, we first genotyped our subjects to identify individuals who are heterozygous for one or more of the *TSC1 *and *TSC2 *marker SNPs described above. The heterozygosities of the *TSC1 *markers rs2809243 and rs739442 were approximately 49% (41/83) and 45% (37/83), respectively, in our sample. Heterozygosity of the *TSC2 *marker SNP rs1748 was approximately 22% (18/82). These data are similar to average population heterozygosities for subjects of all races reported for these SNPs on the SNPper website (CHIP Bioinformatics resource - http://snpper.chip.org) and the NCBI SNP database. Approximately 36% (30/83) of subjects were heterozygous at both *TSC1 *SNPs.

### AEI in the TSC1 gene

AEI analysis of *TSC1 *mRNA expression was performed independently using the marker SNPs rs2809243 and rs739442. As indicated above, 41 individuals were heterozygous at rs2809243 and 37 were heterozygous at rs739442. 30 individuals were heterozygous at both of the marker SNPs. Data from these doubly heterozygous individuals was used for independent validation of the results from each SNP. As outlined in Figure 1, AEI measurements using genomic DNA as template were carried out to permit the calculation of a correction factor for AEI measurements using cDNA as template. AEIs were considered to be significant if the corrected cDNA allelic expression ratio differed from the corrected genomic allele ratio by greater than 10%, and if the error bars (defined here as 2x the standard error of the mean) for the average genomic and cDNA allele ratios did not overlap.

**Figure 3:**
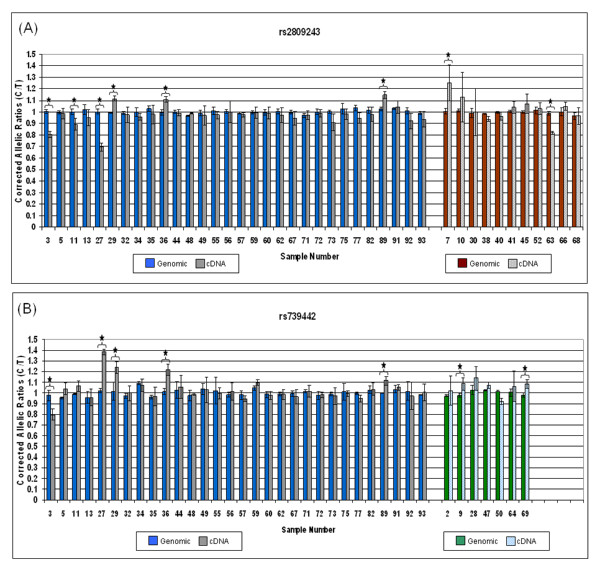
**AEI analysis of TSC1 mRNA expression in leukocytes isolated from 30 individuals doubly heterozygous for the marker SNPs rs2809243 and rs739442 and additional individuals (11 and 7 individuals respectively) singly heterozygous for one of the two SNPs**. For doubly heterozygous individuals (data at the left side of graphs 3A and 3B), blue bars indicate corrected genomic allelic ratios (AR) and grey bars indicate the corrected cDNA ARs. Data for singly heterozygous individuals is located to the right side of the 3A and 3B graphs using red and green bars to indicate genomic ARs and shaded bars to indicate cDNA ARs. Error bars indicate 2X the standard error of the mean (SEM). Stars indicate samples for which the average corrected cDNA AR differed from the genomic AR by more than 10% and had error bars that did not overlap with those of the genomic DNA.

Figure 3 displays the corrected genomic and cDNA AEI ratios for each individual in our sample. Shown to the left in each graph is the data for individuals heterozygous at both marker SNPs. Shown to the right in each graph is additional data for individuals heterozygous at a single marker SNP. 8/41 individuals show AEI using rs2809243 while 7/37 individuals show AEI using rs739442. Of the doubly heterozygous individuals, rs2809243 revealed 6 individuals with AEI reaching our defined level of significance while rs739442 showed 5 individuals demonstrating AEI. The 5 individuals with AEI by rs739442 were the same as those with AEI by rs2809243. A 6^th ^individual's sample (#11) reached AEI significance by a small margin using rs2809243 but did not reach significance using rs739442 as the marker SNP, thus emphasizing the importance of using a second SNP to validate data. We were able to consistently score 5 out of 6 individuals as demonstrating significant AEI in blood leukocytes using two different SNPs. The AEI of *TSC1 *mRNA expression in this control group ranged from 10% to greater than 30%. While this degree of imbalance is relatively small, it could be sufficient to modulate the phenotype in a TSC patient heterozygous for a mutation in the *TSC1 *gene. Based on this small cohort of control subjects we estimate that the population frequency of AEI at the TSC1 locus may be as high as 15-20%.

It should be noted that the cDNA allelic expression ratios measured using the marker SNP rs280943 range from greater that 1 (samples 29 and 36) to less than 1 (samples 3 and 27). This implies that the regulatory variant or variants resulting in this AEI are not tightly linked to the marker SNP. Thus, if these individuals were heterozygous for a remote regulatory variant comprising one high-expression allele and one low-expression allele, the results of our AEI measurements imply that the high-expression allele is "in phase" (ie., located on the same chromosome) with the rs280943 C-allele in individuals 29 and 36, but is "in-phase" with the rs280943 T-allele in individuals 3 and 27.

Similar arguments hold for the AEI measurement obtained using rs739442 as the marker SNP. The fact that the directions of the measured AEI differ for individual #27, depending upon the choice of marker SNP, implies that the "phase" of the marker SNPs with respect to the functional variant is different in this individual. That is, in this individual the rs739442 C-allele is located on the same chromosome as the high-expression allele of the remote regulatory variant. Although the two SNPs used for these analyses (rs280943 and rs739442) are separated by only 166 bp, our data indicate that these SNPs are not tightly linked. As previously indicated, only around 29% of our sample population is heterozygous at both *TSC1 *marker SNPs despite their close proximity. This is apparent in the data of individual #27 which shows the marker SNPs to be on different chromosomes. Using the Hapmap database http://hapmap.ncbi.nlm.nih.gov the linkage disequilibrium D' value for these SNPs is 0.671, confirming that these SNPs are not tightly linked despite the small separation distance. As these two SNPs are both located in the 3'UTR of *TSC1*, it is not surprising to see this level of variation as mutations in this area are less likely to affect the protein function.

### AEI in the TSC2 gene

**Figure 4:**
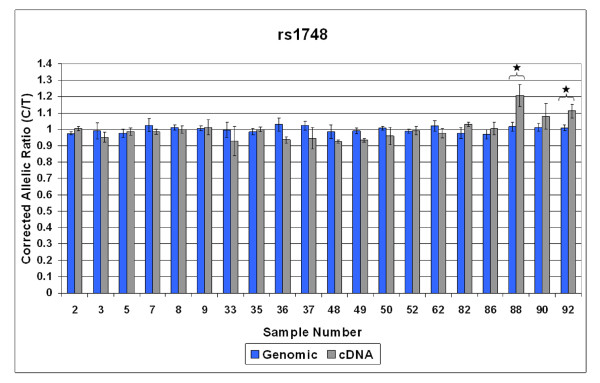
**AEI analysis of TSC2 mRNA expression in leukocytes isolated from 20 individuals heterozygous for the marker SNP rs1748**. Blue bars indicate corrected genomic allelic ratios (AR) and grey bars indicate corrected cDNA ARs. Error bars indicate 2X the standard error of the mean (SEM). Stars indicate samples for which the average corrected cDNA AR differed from the average corrected genomic AR by more than 10% and had error bars (2X SEM) that did not overlap with those of the genomic DNA.

Twenty out of 83 individuals in our sample were heterozygous for the *TSC2 *mRNA marker SNP rs1748, As shown in Figure 4, 10% (2/20) of these individuals demonstrated AEI above the 10% cut-off, with a difference of more than 2x the SEM. An independent marker SNP was not available for verification; however, AEI measurements were highly reproducible. Our data demonstrates that AEI is relatively common in both the *TSC1 *and TSC2 genes.

## Discussion

There is a growing consensus that *cis*-acting genetic variants significantly contribute to phenotypic differences among individuals, including disease risk [36,39,41-45]. Regulatory polymorphisms are one of the predominant mechanisms by which *cis*-acting gene regulation has been found to occur. These polymorphisms, located in regulatory regions, influence the expression of genes by affecting transcriptional activation or repression, generally by altering the DNA binding sites for transcription factors [36,39,46,47]. In addition, splicing errors, changes in mRNA stablility, epigenetic modifications and polymorphic (AC_n_) microsatellites have also been implicated in cis-acting gene regulation [36,37,39,46-49].

The best known examples of allele-specific differences in gene expression have been associated with X-inactivation [50] or genomic imprinting [51]. However, allelic variation in expression has also been demonstrated in non-imprinted genes and this allelic variation itself can be regulated by cis-acting elements [36-39]. Variations in allele expression have been previously linked to disease. For example, allelic variation in APC (adenomatous polyposis coli) expression plays a role in predisposition to colon cancer [41]. Allelic expression imbalance has also been studied in the cancer associated genes *BRCA1/2 *and *CDH1 *and used to identify polymorphisms, mutations and other defects that alter allelic expression and influence disease state [45,48]. As many genes are active within networks, variation in the expression of specific gene alleles may ultimately result in multiple downstream effects within a network and between related gene networks. This creates an avenue by which even small differences in the expression of specific genes can ultimately result in substantial phenotypic changes [46,47].

It has been noted frequently that disease causing mutations in families with TSC may produce very few problems in certain individuals while having catastrophic effects in others [2,26-31]. Clearly, there are additional factors outside of the mutation itself that affect disease severity. Differences in allele specific mRNA expression could potentially be one of these disease modifying factors. It is possible that the overall amount of normal TSC protein in cells may determine the severity of the disease phenotype in patients. As TSC is a disease carried in a heterozygous state [1-3], some amount of normal TSC protein should be present in most cells since one normal allele of each TSC gene is present (the exception being abnormal tissue growths exhibiting LOH [15-18]). It is possible that higher relative expression of protein from the normal allele may be protective, while higher relative expression of abnormal protein from the mutant allele may have deleterious effects.

We began to study this issue by determining the frequency of occurrence of AEI of the *TSC1 *and *TSC2 *genes in a control population. The intent was to establish if mRNA expression variation might be common enough to be a mechanism by which phenotypes are modified in patients with TSC gene mutations. In a cohort of normal volunteers we were able to quantify allele specific expression of the TSC genes in blood RNA and estimate the frequency of allelic skewing of expression for these two genes. In our studies we found that there was significant skewing of allelic expression of the *TSC1 *gene in about 19% of our sample population and of the *TSC2 *gene in 10% of our population. This estimate is based on a small sample of informative individuals (48 individuals who were heterozygous for a TSC1 marker SNP and 20 who were heterozygous for a TSC2 marker SNP). This was a sample of convenience, but individuals were recruited without bias and should be representative of the general population. If we assume a binomial distribution for the occurrence of AEI in the general population, we can use the exact test to calculate 95% confidence intervals for the actual population frequencies of AEI at the TSC1 and TSC2 genes. Based on such calculations, the 95% confidence interval for prevalence of AEI at TSC1 is 9% to 33% and for AEI at the TSC2 gene is 1.2% to 32%. These confidence intervals for the estimates for the actual population frequencies of AEI at TSC1 and TSC2 can of course be sharpened with larger sample sizes.

While these are not large proportions, AEI may be occurring frequently enough to be a potential contributor to the phenotypic differences in TSC patients. In any given individual patient within a particular family, the phenotype could be determined not just by the mutation, but also by SNPs located within regulatory regions of the TSC genes. In such familial cases of TSC, the implication is that regulatory SNPs inherited from the parents in various combinations with the normal and mutant gene alleles can affect the phenotype of the child.

The *TSC1 *and *TSC2 *gene products, hamartin and tuberin, function together as a protein complex. Therefore, mutation of either of the TSC genes results in the same disease [1,3,52]. The hamartin-tuberin complex is a modulator of the mTOR signaling pathway, which is important in the regulation of cell growth. We know that haploinsufficiency due to mutation of a single TSC gene allele is sufficient to cause TSC and represents an approximately 50% loss of the total expression of that TSC gene [2]. Loss of a single TSC gene allele is sufficient to disrupt neuronal morphology and function in mouse models [21]. Loss of both alleles of a TSC gene can result in the formation of hamartomas common to TSC as is demonstrated by LOH studies [13,15-18]. These points clearly suggest that pathways modulated by hamartin and tuberin are sensitive to gene dosage effects. If a 50% reduction in expression of a TSC gene is sufficient to cause disease, it is plausible that smaller variations in expression, such as the 10-30% that we found in our experiments, might be sufficient to influence phenotype. In our control sample group, this level of variation in allelic expression of *TSC1 *or *TSC2 *does not result in a phenotype, as both alleles encode normal proteins. This degree of variation in mRNA expression combined with mutation of a TSC gene allele may be sufficient to influence phenotype either positively or negatively.

It has previously been reported that a 50% decrease in the expression of a single allele of the adenomatous polyposis coli tumor suppressor gene (*APC*), representing an overall 25% decrease of APC mRNA expression, is sufficient to cause the development of familial adenomatous polyposis[41]. An additional study of a gene associated with osteoarthritis (*GDF5*) discovered that a promoter polymorphism which created a small reduction of the expression of the T allele (less than 27%), significantly increased individuals susceptibility to developing osteoarthritis [53]. These reports indicate that even small variations in allelic expression are important to disease outcomes. This supports our hypothesis that variation in expression of the TSC gene alleles, particularly in the presence of an existing genetic mutation, may influence disease severity in TSC patients.

Tissue specific expression of genes is an important consideration when assessing the effects of variation in allelic expression. A sequence variant in the regulatory region of a gene might be relevant in some tissues and not in others, leading to conflicting results in different tissues [54]. Our study was performed in blood samples as this is a readily available tissue specimen. It is important to determine if allelic expression ratios measured in peripheral blood correlate with ratios measured in brain tissue, something that may be done using banked tissue samples. A difficulty we've encountered is the availability of good-quality matched blood and brain tissue samples from which intact RNA and DNA can be extracted. Establishing a correlation between blood and brain expression levels is especially important as we try to relate expression of the *TSC *alleles in blood to severity of cognitive impairment in patients with TSC. The goal of our research is to determine if the levels of expression of mutant and wild-type alleles in patients with TSC, as measured in peripheral blood, correlates with phenotypic severity. To this end, we plan to next study familial cases of TSC, where multiple affected individuals have the same identical gene mutation, but are discordant in terms of disease severity. We shall determine if, in these multiplex families, disease severity is correlated with skewing of allele specific expression. Ultimately, we hope to use the combination of mutation detection and measures of AEI in blood samples to predict disease severity (at least in relation to cognitive impairment). Early identification of patients who are at risk for developing severe disease may allow for aggressive preventive interventions, and may protect the patient from additional damaging effects of the disease.

## Conclusions

We have concluded from our research that variation in *TSC1 *and *TSC2 *gene allele expression is common in normal individuals, as it was easily detected in a relatively small sample population. It is likely that this variation in allele expression will also be seen in some patients carrying TSC gene mutations and may therefore help to explain the intra-familial variation in disease severity frequently observed in TSC. These ideas can be tested in multiplex families that include patients with TSC that are discordant in disease severity (particularly cognitive symptoms). After such validation, we might be able to develop a simple blood test (ratio of wild-type to mutant TSC mRNA levels) that predicts disease severity in simplex cases of TSC.

## Competing interests

The authors declare that they have no competing interests.

## Authors' contributions

VN conceived of the study and VN, GJ and SR participated in its design and coordination. SR performed the DNA/RNA isolation and the AEI analysis. SO performed DNA sequencing and genotyping and provided theoretical advice. GJ and VN drafted the manuscript and SR and SO edited the manuscript. DS provided key technical advice and critical review of the manuscript. All authors read and approved the final manuscript.

## Pre-publication history

The pre-publication history for this paper can be accessed here:

http://www.biomedcentral.com/1471-2350/12/29/prepub
